# HIV and Associated Indicators of COVID-19 Cytokine Release Syndrome: A Meta-Analysis and Meta-Regression

**DOI:** 10.7759/cureus.34688

**Published:** 2023-02-06

**Authors:** John K Muthuka, Kelly Oluoch, Francis M Wambura, Japheth M Nzioki, Rosemary Nabaweesi

**Affiliations:** 1 Epidemiology, Public Health & Biostatistics, Jomo Kenyatta University of Agriculture & Technology, Nairobi, KEN; 2 Public Health Sciences, Kenya Medical Training College, Nairobi, KEN; 3 Pharmacy, Kenya Medical Training College, Nairobi, KEN; 4 Public Health, Kenya Medical Training College, Nairobi, KEN; 5 College of Health Sciences, Jumeira University, Dubai, ARE; 6 Health Policy, Meharry Medical College, Nashville, USA

**Keywords:** coronavirus disease 2019, systematic review and meta-analysis, cytokine release syndrome (crs), hiv aids, infectious diseases epidemiology, epidemiology and biostatistics

## Abstract

The aim of this review was to evaluate the risk of COVID-19 cytokine release syndrome (CRS) with HIV infection and meta-regress for indicator covariates. Electronic databases, including Google Scholar, Cochrane Library, Web of Sciences (WOS), EMBASE, Medline/PubMed, COVID-19 Research Database, and Scopus, were systematically searched till February 30, 2022. All human studies were included, irrespective of publication date or region. Eleven studies, with a total of 2,005,274 detailing cytokine release syndrome defined by specific parameters, were included. To pool the estimate, a random-effects model with risk ratio (RR) as the effect measure was used. Moreover, publication bias and sensitivity analysis were evaluated followed by meta-regression analysis to account for any possible covariates. This systematic review, meta-analysis, and meta-regression trial was registered (CRD42021264761) on the PROSPERO register. HIV infection showed an increased risk for COVID-19 cytokine release syndrome (RR= 1.48, 95% CI (1.16, 1.88) (P=0.002)) with substantial heterogeneity (I^2^ > 80%) and a 4.6% cumulative incidence. The true effects size in 95% of all the comparable populations (prediction interval) fell between 0.67 to 3.29. HIV infection further showed an increased risk for intensive care unit (ICU) admission ((P<0.0001) (I² = 0%)] and mechanical ventilation (MV) ((P=0.04) (I² = 0%)) as the key indicators of cytokine release syndrome. Meta-regression analysis demonstrated that COVID-19 cytokine release syndrome was influenced by the year a study was published (R² = 0.55) and the region from where the study was conducted (R² = 0.11). On meta-regression analysis, the combined impact of all covariates in the model explained at least some of the variance in effect size (Q = 16.21, df = 6, P= 0.0127), and the proportion of variance explained by covariates on comparing the model with and without the covariates was 73 % and highly significant (Tau² = 0.1100, Tau = 0.3317, I² = 86.5%, Q = .99, df = 10, P<0.0001) (R² = 0.73). Our updated meta-analysis indicated that HIV infection was significantly associated with an increased risk for COVID-19 cytokine release syndrome, which, in addition, might be moderated by the year a study was published and the region in which the study was conducted. Further, the risk for intensive care unit (ICU) admission and mechanical ventilation (MV) were identified as the key indicators of cytokine release syndrome. We believe the updated data anchoring cytokine release syndrome will contribute to more substantiation of the findings reported by similar earlier studies.

## Introduction and background

About 38 million people living with HIV (PLWH) globally, including 1.7 million children, with a global HIV prevalence of 0.7% among adults [1], may have an increased risk of adverse outcomes from coronavirus disease 2019 (COVID-19) infection as a result of HIV-associated immune dysfunction due to the associated cells’ alterations and depletion [2]. There may also be a higher prevalence of comorbidities among PLWH that predispose them to adverse COVID-19 outcomes [3]. Conversely, PLWH may have more favorable outcomes due to increased health awareness or close medical follow-up and constant reviews with some specific antiretroviral agents under consideration as potential treatments for COVID-19 [4].

Severe COVID-19 disease manifested by fever and pneumonia, leading to acute respiratory distress syndrome (ARDS) has been described in up to 20% of COVID-19 cases. This is reminiscent of cytokine release syndrome (CRS)-induced ARDS and secondary hemophagocytic lymph histiocytosis (sHLH) observed in patients with SARS-CoV-2 [5], characteristics of CRS, including pulmonary inflammation, fever, and dysfunction of non-pulmonary organs. An increase of interleukin-6 in peripheral blood is a key risk factor and an early indicator of CRS in COVID-19. Both antibody and T-cell responses are critical for the effective control and clearance of SARS-CoV-2. More severe COVID-19 disease correlates with lymphopenia and low T-cell concentrations [6].

COVID-19-associated CRS by HIV serostatus is not explicitly researched and most meta-analyses have focused on studies lacking comparator groups or they used a general population as controls unlike in the current study, which restricts the comparator as HIV negative in the same included study. The study aimed at evaluating the evidence on the risk of COVID-19 CRS in PLHIV using both earlier and recently published data, and a meta-regression to ascertain the extent to which this risk is modified by other possible covariates.

A portion of the content of this current article was previously posted to the Multidisciplinary Preprint Platform server under Preprints on COVID-19 and SARS-CoV-2 on May 13, 2022.

## Review

Materials & methods

Study Design and Search Strategy

We utilized a systematic review to identify studies between April 1, 2020, and February 30, 2022, which described cytokine release syndrome in people living with HIV (PLWH) and compared them with HIV-negative people, and a meta-analysis approach, followed by a meta-regression, to ascertain the covariates associated with COVID-19 cytokine release syndrome.

A standard search strategy was used in electronic databases, including Google Scholar, Cochrane Library, Web of Sciences (WOS), EMBASE, Medline/PubMed, COVID-19 Research Database, and Scopus, and then modified according to each specific database to get the best relevant results. These included Medline-indexed journals; PubMed Central; NCBI Bookshelf, medRxiv, Lit Covid, Trip, Google, Google Scholar, and publishers' Web sites. The basic search strategy was built based on the research question formulation (i.e., PICO or PICOS; in the context of this review, P, the study population being COVID-19 infected subjects, I, the intervention being the HIV infection, C, the comparison being HIV seronegative status and O, the outcome being the COVID-19-related cytokine release syndrome). They were constructed to include free-text terms (e.g., in the title and abstract) and any appropriate subject indexing (e.g., MeSH) expected to retrieve eligible studies, with the help of an expert in the review topic field or an information specialist. The summary of search terms was; COVID-19 severity; Corona Virus Severity; Cytokine Storm, Cytokines; HIV; Inflammation; Chemokine; Interleukins and immune reactions, COVID-19 mortality, etc. After some rounds of trial, refinement, and formulation of the search terms for PubMed as follows: (COVID-19 OR corona-virus virus OR coronavirus disease) AND (“the study” [Publication Type] OR “study as the topic” [MeSH Terms] OR “study” AND HIV serostatus AND [All Fields]). One author with extensive literature search experience and expertise performed the preliminary screening to exclude duplicates and studies not related to HIV infection. For the remaining articles, another author performed title/topic and abstract screening, with subsequent full-text review by two authors using a standardized data extraction form. Where disagreement was feasible, inclusion decisions were made by a third author. We also included preprints to capture the most recent and emerging evidence. Studies with 15 or fewer participants were excluded, as they were less likely to have the power to detect meaningful relationships. The quality of the studies was evaluated using the Newcastle-Ottawa Scale for observational studies [7].

Studies included, effect measures, and analysis

Observational studies reporting any possible indicator of COVID-19-related cytokine release syndrome in people with and without HIV were included in a meta-analysis. Specific relative risks (RRs) and hazard ratios were combined with a random effects model to account for the variability of the true effect between studies. To explore possible effect modifications, subgroup and meta-regression analyses were conducted for COVID-19-related CRS. Meta-analysis was performed in RevMan 5.4 (Review Manager. (RevMan) [Computer program]. Version 5.4. The Cochrane. Collaboration, 2020) and CMA-v3 (dichotomous data, random effects model) calculated the effect estimates as risk ratios (with 95% CI).

 Study Selection Procedure

**Figure 1:**
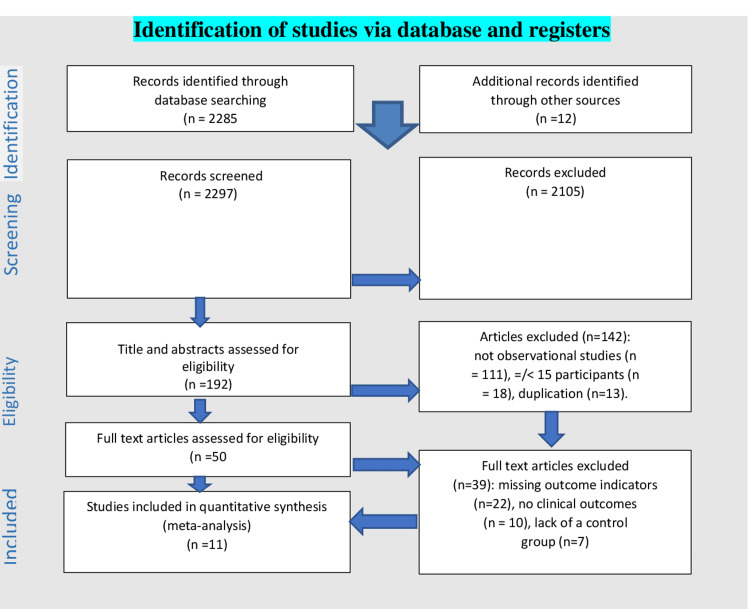
PRISMA 2022 Flow Diagram PRISMA: Preferred Reporting Items for Systematic Reviews and Meta-Analysis

We identified 2285 records and included a total of 11 studies detailing cytokine storm syndrome as an outcome in our final analysis. The included studies were peer-reviewed, with some as preprints since the research quest sought to capture even the latest data and information. The 11 studies [8-18] reported and compared cytokine storm syndrome, defined by a specific parameter (such as intensive care unit admission) between HIV seropositive and seronegative persons. The procedure is shown in Figure 1.

Quality of evidence and risk of bias assessment

We assessed the quality of the included studies based on a modified version of the Newcastle-Ottawa Scale (NoS), which consists of eight items with three sub-scales, and the total maximum score of these three subsets is 9. We considered a study that scored ≥7 a high-quality study since a standard criterion for what constitutes a high-quality study has not yet been universally established. The studies assessed generated a mean value of 6.59 and as a result, the overall quality was found to be moderate (NOS score min: 5, max: 8). There were common limitations among the included studies. Most were retrospective analyses of routinely collected clinical data, meaning the identification of COVID-19 cases was not systematic and depended on the local approach to screening and diagnosis with only five prospective cohort studies. This may have varied over time and between settings and may also differ between PLWH and the general population but in the case of this study though, the studies included both HIV-seropositive and seronegative populations’ data. Across all studies, the numbers of HIV-seropositive and COVID-19 infections were relatively low. The Newcastle Ottawa Scale (NoS) information is shown (Table 1) [17].

**Table 1:** Quality of evidence and risk of bias assessment Newcastle Ottawa Scale (NoS) consisting of eight items with three sub-scales and a total maximum score of 9 The asterisk(*) numbers here depict the level of agreement in terms of each author's view. Using the tool, each study is judged on eight items, categorized into three groups: the selection of the study groups; the comparability of the groups; and the ascertainment of either the exposure or outcome of interest for case-control or cohort studies, respectively. Stars awarded for each quality item serve as a quick visual assessment. Stars are awarded such that the highest quality studies are awarded up to nine (9) stars.

Study	Case selection (max. 4)	Comparability (max. 2)	Exposure/outcome (max. 3)	Total score
[15]	***	**	**	7
[9]	***	*	**	6
[13]	****	**	**	8
[11]	***	**	***	7
[16]	***	**	**	7
[8]	***	**	**	7
[12]	***	**	*	6
[10]	***	**	**	6
[18]	***	*	***	7
[17]	***	*	**	6
[14]	**	***	*	6

Results

In this meta-analysis pool, 2,005,274 from 11 studies [8-18] with cytokine release syndrome diagnosed with COVID-19 were included utilizing the predefined given Centers for Disease Control and Prevention (CDC) reporting guidelines on COVID-19 diagnosis [19]. The cumulative COVID-19 cytokine release syndrome defining parameter was 48863 (2.4%). The total COVID-19-related CRS was 837(4.6%) and 48026 (2.4%) among the HIV seropositive and HIV-seronegative persons, respectively. The cumulative incidence of COVID-19-related cytokine release syndrome ranged from 1.5% to 40 % (average: 19 %). A summary of the studies included in this meta-analysis is available (Table 2).

**Table 2:** A summary of the studies included in this meta-analysis CRS: Cytokine Release Syndrome

Study	Region	Study Design & Setting	CRS in PLWH	CRS in Non-HIV People	Cumulative %
[8]	United States of America	Retrospective Cohort, Multiple	124 / 2419	6060 / 202012	3.024982
[12]	United States of America	Prospective Cohort, Multiple	78 / 404	5264 / 49763	10.64843
[10]	Spain	Retrospective Cohort, Multiple	2 / 21	24 / 105	20.63492
[14]	United States of America	Retrospective Cohort, Multiple	6 / 21	10 / 42	25.39683
[15]	United States of America	Retrospective Cohort, Multiple	475 / 13158	24579 / 1420751	1.747252
[9]	United States of America	Retrospective Cohort, Single	5 / 10	494 / 1976	25.12588
[11]	United States of America	Prospective Cohort, Multiple	59 / 220	6545 / 21319	30.66066
[16]	United States of America	Retrospective Cohort, Multiple	57 / 1629	4297 / 286467	1.511302
[13]	United States of America	Retrospective Cohort, Single	21 / 100	631 / 4513	14.13397
[17]	Israel	Retrospective Cohort, Single	2 / 23	103 / 254	37.90614
[18]	United Kingdom	Retrospective Multi-Center	8 / 17	19 / 50	40.29851
Total Cumulative					48863/ 2005274 (2.4367%)

Risk of Cytokine Release Syndrome With HIV Infection

**Figure 2:**
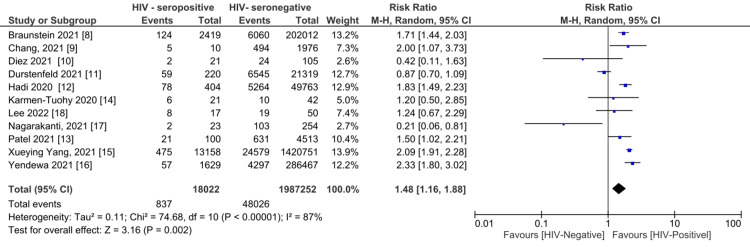
Forest plot demonstrating pooled CRS for HIV-positive serostatus compared to HIV-negative serostatus Note: Weights are from Mantel-Haenszel (M-H) model, random effects (RE) at 95% C.I.

**Figure 3:**
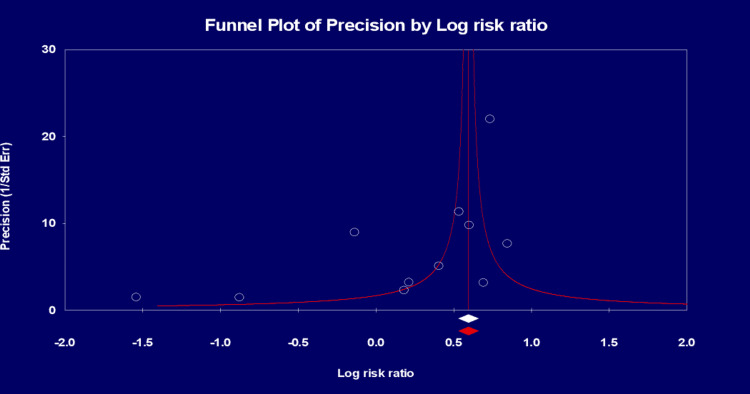
Precision funnel plot analysis A precision funnel plot supplementing Egger’s regression intercept test for a publication bias.

From the 11 studies [8-18], a total of 48,863 (2.4%) patients experienced cytokine release syndrome (CRS). The analysis demonstrated a 48% increased risk of CRS with HIV seropositive status (risk ratio = 1.48, 95% confidence interval (CI) (1.16, 1.88) (P = 0.002) (Figure 2) and a considerable true heterogeneity (I^2^) between all the pooled studies (I² = 87 %; P<0.0001). A precision funnel plot with Egger’s regression intercept test indicated a publication bias (intercept = -2.23097, 95% confidence interval (-4.76459, 0.30266), with t=1.99193, df=9. The 1-tailed P = 0.03878). The precision funnel plot subject to the forest plot was obtained (Figure 3).

**Figure 4:**
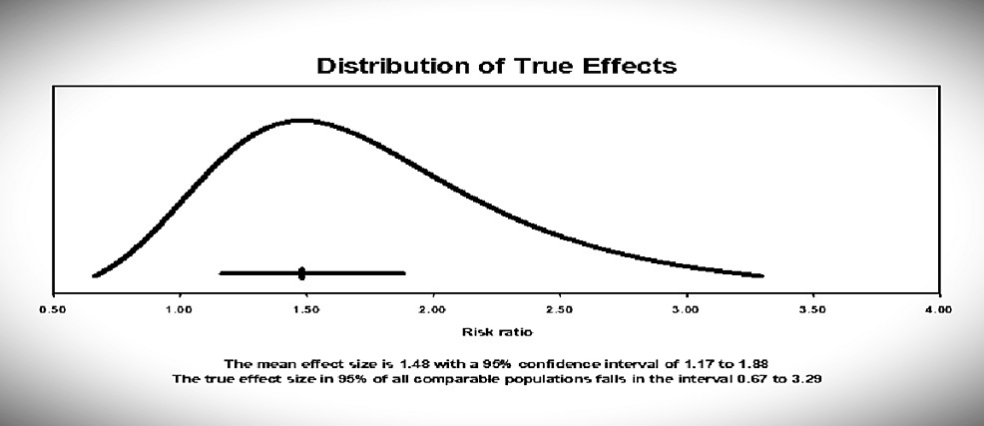
Distribution of true effects The prediction interval of the true effects size in 95% of all the comparable populations using the random effects model. The mean effect size is 1.48 with a 95% confidence interval of 1.17 to 1.88 while the true effect size of all comparable populations falls in the interval 0.67 to 3.29.

The prediction interval demonstrated the true effects size in 95% of all the comparable populations falling between 0.67 to 3.29, which depicted that, in some populations, the risk of COVID-19 cytokine release syndrome due to HIV infection is at one extreme of effect as low as 0.67 and as high as 3.29, thus necessitated accounting for any possible covariates (Figure 4).

**Figure 5:**
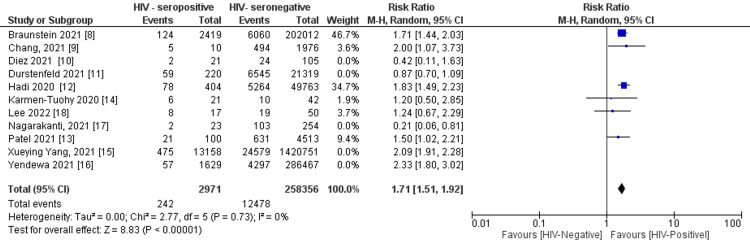
Pooled CRS sensitivity analysis on HIV serostatus using random effects model (R.E.) CRS: Cytokine Release Syndrome, M-H: Mantel-Haenszel model

**Figure 6:**
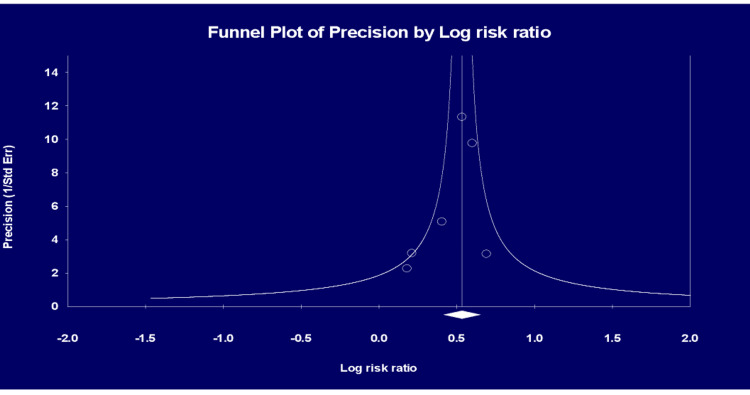
Precision funnel plot on sensitivity with the remaining six studies

Sensitivity analysis by removing five studies [10,11,15-17], which caused major heterogeneity, explicitly showed a risk of CRS with HIV seropositive (risk ratio = 1.71, 95% confidence interval (CI) (1.51, 1.92) (P < 0.0001) (I² = 0%)), with no-publication bias as revealed by the funnel precision plot in a total of 12720 (4.9%) (Figure 5). The publication bias test revealed by the precision funnel plot clearly showed non-publication bias in a total of 12720 (4.9%) on the six remaining studies [8,9,12-14,18] (Figure 6).

Sub-Group and Sensitivity Analysis on CRS Indicator by HIV- Serostatus

**Figure 7:**
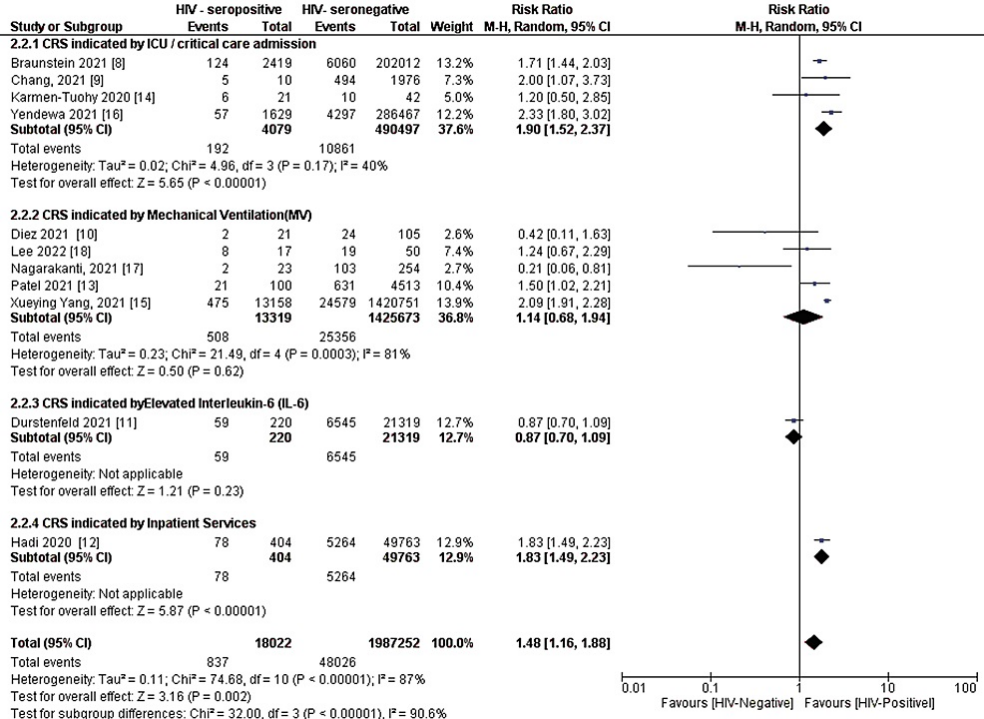
Pooled CRS subgroup analysis by specific indicators (parameters) Note: Weights and the between-subgroup heterogeneity tests are from the Mantel-Haenszel model. CRS: Cytokine Release Syndrome

In the context of this study, CRS was implicated by critical care services (ICU) admission in four studies [8,9,14,16], mechanical ventilation in three studies [10,17,18], increased intubation rates in one study [13], elevated interleukin-6 in one study [11], clinical severity of COVID-19 in one study [15], and needed inpatient services in one study [12]. On subgroup analysis, HIV seropositive status showed a risk for ICU/critical care service (Rrsk ratio = 1.90, 95% confidence interval (CI) (1.52, 2.37) (P < 0.0001) (I² = 40%)) and general inpatient services (risk ratio = 1.83, 95% confidence interval (CI) (1.49, 2.23) (P < 0.0001)), but not with elevated interleukin-6 (IL-6) (P = 0.23) and mechanical ventilation (Risk ratio = 1.14, 95% confidence interval (CI) (0.68, 1.94) (P = 0.62) (I² = 81%)). Test for subgroup differences had high heterogeneity (I² = 90.6%) (Figure 7).

**Figure 8:**
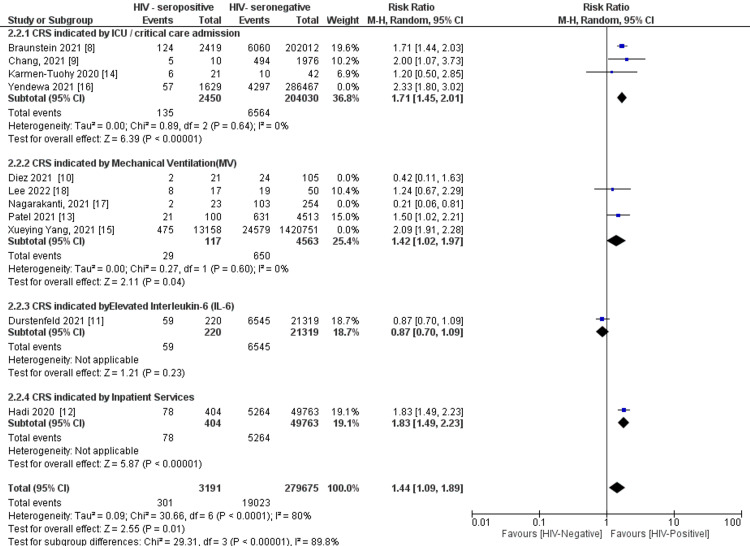
Sensitivity analysis on CRS indicator by HIV- serostatus Note: Weights and the between-subgroup heterogeneity test are from the Mantel-Haenszel model. CRS: Cytokine Release Syndrome

Further, sensitivity analysis on subgroups clearly demonstrated that HIV seropositive status had a risk for CRS indicated by intensive care unit (ICU) admission (P < 0.0001) (I² = 0%) after removing one study [16] and mechanical ventilation (P = 0.04) (I² = 0%) after removing three studies causing major between study differences [10,15,17] (Figure 8).

Meta-Regression for Possible Moderators of COVID-19 CRS With HIV- Serostatus

The values of heterogeneity (I^2^) in studies accounting for COVID-19 CRS was 87, which means the observed variance came from real differences between studies and, as such, can potentially be explained by study-level moderators. Hence, the analysis assessed the possible influence of predetermined moderators. On the test of the individual covariate, the period of the year a study was conducted predicted CRS (Q=10.63, df=4, P = 0.0311). Studies conducted after June 2020 (June > 2020) were more significant (P = 0.0458) (R² = 0.70) (Supplementary File 1), the study setting (single or multiple sites) was insignificant (P = 0.4576) (R² = 0.06) (Supplementary File 2), and the region/country of the study population (Q = 5.83, df = 1, P = 0.0158) (R² = 0.20) (Supplementary File 3). The combined impact of all covariates in the model explained at least some of the variance in effect size (Q = 16.21, df = 6, P = 0.0127), and the proportion of variance explained by covariates on comparing the model with and without the covariates was 73% and very significant (Tau² = 0.1100, Tau = 0.3317, I² = 86.5%, Q = .99, df = 10, P < 0.0001) (R² = 0.73) (Supplementary File 4).

Discussion

The purpose of this study was to systematically review and conduct a meta-analysis using the most current data from studies on the incidence of COVID-19-related cytokine release syndrome relative to HIV serostatus, alongside the associated covariates via meta-regression. Further, it aimed at ascertaining the parameters defining cytokine release syndrome predicted by HIV infection and estimating the combined proportion effect of all covariates in studies detailing CRS.

Principally, the present meta-analysis found that HIV seropositive status was significant in predicting CRS by over 50%. Following sensitivity analysis of good-quality studies only, the risk for both COVID-19-related CRS was more significant. Overall, there was a high degree of heterogeneity among studies detailing COVID-19-related CRS, which greatly reduced following sensitivity analysis. The outcome remained significant on the inclusion of only good-quality studies suggesting these analyses represent true effects as per the generated prediction intervals. A high level of heterogeneity was only observed with the inclusion of a few studies in assessing the effect of HIV on COVID-19-related CRS, likely to substantial inter-study variation. Egger’s regression test indicated a low impact of publication bias on our results.

The finding that HIV clearly predicts more significantly the experience of cytokine release syndrome confirms previous findings [20]. The association of CRS with HIV seropositive status in the context of this current findings is biologically plausible as in normal circumstances, CRS is linked with acute respiratory distress syndrome (ARDS), which leads to COVID-19 severity prior to case fatality (death) [21].

CRS indicators were critical care services (ICU) admission, mechanical ventilation, increased intubation rates, elevated interleukin-6, clinical severity of COVID-19, and inpatient services. These trends are similar to other studies that demonstrated that HIV infection is associated with ICU admission, mechanical ventilation [14], intubation [22], interleukin-6 [23], and clinical severity of COVID-19 inpatient services [24]. In this current study, mechanical ventilation and ICU admission clearly showed an association with HIV seropositive status with a similar trend of increased risk [14], but this is contrary to another study that found no difference in HIV infection and non-infection [17].

Meta-regression analysis showed that the year (2020, 2021, and not 2022) and the region in which a study was conducted were associated with COVID-19-related cytokine release syndrome (P < 0.05), unlike the study setting sites. Generally, the combined impact of all covariates in the model explained at least some of the variance in COVID-19-related CRS, similar to existing findings in countries and region-related factors [25].

Some limitations were noted in our review and meta-analysis. The included studies did not put into categories clear HIV infection staging as per the WHO criteria [26], thus it made it impossible to conduct a subgroup analysis on PLWH based on that. Cytokine release syndrome is multifaceted and an acute systemic inflammatory syndrome characterized by fever and multiple organ dysfunction that is associated with chimeric antigen receptor (CAR)-T cell therapy [27], however, the study focused on the clinical outcomes that were defined by specific parameters such as ICU admission, though with the clear presumption that this would ensue due to the pathophysiology of the cytokine release syndrome [28].

## Conclusions

Our study indicated a consistent and statistically significant effect of HIV on COVID-19-related cytokine release syndrome even after heterogeneity investigation all in the random effects model with Egger’s intercept regression test indicating no major publication bias. ICU admission, mechanical ventilation, and intubation were the key CRS parameters predicted by HIV infection in COVID-19 patients. The proportion of variance explained by covariates was significant with the year a study was conducted, the region of the study population, and the study setting, either single or multiple center, being the major covariates associated with COVID-19-related CRS.

Public health interventions should be carefully tailored and implemented on PLWH and infected COVID‐19 to reduce the risk of severity associated with cytokine release syndrome, a key predictor of COVID-19 case fatality. An intensive and regular focus is required to detect early occurrences of clinical conditions in similar viral pandemics or COVID-19 resurgence.

## Appendices

**Table 3:** Supplementary File 1 Meta-regression analysis on the period the study was conducted

	Main results for Model 1, Random effects (ML), Z-Distribution, Log risk ratio											
Set	Covariate	Coefficient	Standard	95%	95%	Z-value	1-sided		
			Error	Lower	Upper		P-value	Set	
	Intercept	0.6017	0.2083	0.1934	1.0099	2.89	0.0019		
Period of the year a study was conducted		Period of the year a study was conducted: < June 2020				-0.3151	0.2677	-0.8398	0.2096	-1.18	0.1196	Q=10.63, df=4, p=0.0311	
Period of the year a study was conducted		Period of the year a study was conducted: < June 2021				0.1339	0.2799	-0.4146	0.6824	0.48	0.3162	Q=10.63, df=4, p=0.0311	
Period of the year a study was conducted		Period of the year a study was conducted: January - December 2020				0.2454	0.3056	-0.3537	0.8444	0.8	0.211	Q=10.63, df=4, p=0.0311	
Period of the year a study was conducted		Period of the year a study was conducted: June > 2020				-0.4266	0.2529	-0.9224	0.0691	-1.69	0.0458	Q=10.63, df=4, p=0.0311	
	Statistics for Model 1											
	Test of the model: Simultaneous test that all coefficients (excluding intercept) are zero											
	Q = 10.63, df = 4, p = 0.0311											
	Goodness of fit: Test that unexplained variance is zero											
	Tau² = 0.0329, Tau = 0.1813, I² = 82.4%, Q = 34.13, df = 6, p = 0.0000											
	Comparison of Model 1 with the null model											
	Total between-study variance (intercept only)											
	Tau² = 0.1100, Tau = 0.3317, I² = 86.5%, Q = 73.99, df = 10, p = 0.0000											
	Proportion of total between-study variance explained by Model 1											
	R² analog = 0.70											

**Table 4:** Supplementary File 2 Meta-regression analysis on the study setting

	Main results for Model 1, Random effects (ML), Z-Distribution, Log risk ratio									
Set	Covariate	Coefficient	Standard	95%	95%	Z-value	1-sided
			Error	Lower	Upper		P-value
	Intercept	0.2454	0.2321	-0.2095	0.7003	1.06	0.1452
	Study Site: Multiple				0.2024	0.2725	-0.3317	0.7366	0.74	0.2288
	Statistics for Model 1									
	Test of the model: Simultaneous test that all coefficients (excluding intercept) are zero									
	Q = 0.55, df = 1, p = 0.4576									
	Goodness of fit: Test that unexplained variance is zero									
	Tau² = 0.1039, Tau = 0.3224, I² = 87.3%, Q = 70.64, df = 9, p = 0.0000									
	Comparison of Model 1 with the null model									
	Total between-study variance (intercept only)									
	Tau² = 0.1100, Tau = 0.3317, I² = 86.5%, Q = 73.99, df = 10, p = 0.0000									
	Proportion of total between-study variance explained by Model 1									
	R² analog = 0.06									

**Table 5:** Supplementary File 3 A meta-regression analysis of the region of the study population

	Main results for Model 1, Random effects (ML), Z-Distribution, Log risk ratio										
Set	Covariate	Coefficient	Standard	95%	95%	Z-value	1-sided	
			Error	Lower	Upper		P-value	Set
	Intercept	0.5025	0.1212	0.265	0.7399	4.15	0	
	Study Region/Country: Other				-0.8588	0.3558	-1.5563	-0.1614	-2.41	0.0079	
	Statistics for Model 1										
	Test of the model: Simultaneous test that all coefficients (excluding intercept) are zero										
	Q = 5.83, df = 1, p = 0.0158										
	Goodness of fit: Test that unexplained variance is zero										
	Tau² = 0.0875, Tau = 0.2959, I² = 86.1%, Q = 64.61, df = 9, p = 0.0000										
	Comparison of Model 1 with the null model										
	Total between-study variance (intercept only)										
	Tau² = 0.1100, Tau = 0.3317, I² = 86.5%, Q = 73.99, df = 10, p = 0.0000										
	Proportion of total between-study variance explained by Model 1										
	R² analog = 0.20										

**Table 6:** Supplementary File 4 A meta-regression analysis showing the proportion of variance explained by covariates on comparing the model with and without the covariates

	Main results for Model 1, Random effects (ML), Z-Distribution, Log risk ratio											
Set	Covariate	Coefficient	Standard	95%	95%	Z-value	1-sided		
			Error	Lower	Upper		P-value	Set	
	Intercept	0.9537	0.5542	-0.1325	2.0399	1.72	0.0426		
	Study Site: Multiple				-0.352	0.5167	-1.3647	0.6607	-0.68	0.2478		
	Study Region/Country: Other				-0.7496	0.3394	-1.4149	-0.0843	-2.21	0.0136		
Period of the year a study was conducted		Period of the year a study was conducted: < June 2020				-0.4194	0.5149	-1.4284	0.5897	-0.81	0.2077	Q=9.47, df=4, p=0.0503	
Period of the year a study was conducted		Period of the year a study was conducted: < June 2021				0.1339	0.2681	-0.3917	0.6595	0.5	0.3088	Q=9.47, df=4, p=0.0503	
Period of the year a study was conducted		Period of the year a study was conducted: January - December 2020				0.2454	0.2949	-0.3327	0.8234	0.83	0.2027	Q=9.47, df=4, p=0.0503	
Period of the year a study was conducted		Period of the year a study was conducted: June > 2020				-0.3944	0.2438	-0.8723	0.0834	-1.62	0.0528	Q=9.47, df=4, p=0.0503	
	Statistics for Model 1											
	Test of the model: Simultaneous test that all coefficients (excluding intercept) are zero											
	Q = 16.21, df = 6, p = 0.0127											
	Goodness of fit: Test that unexplained variance is zero											
	Tau² = 0.0297, Tau = 0.1722, I² = 86.1%, Q = 28.77, df = 4, p = 0.0000											
	Comparison of Model 1 with the null model											
	Total between-study variance (intercept only)											
	Tau² = 0.1100, Tau = 0.3317, I² = 86.5%, Q = 73.99, df = 10, p = 0.0000											
	Proportion of total between-study variance explained by Model 1											
	R² analog = 0.73											
